# Gi/o-coupled muscarinic receptors co-localize with GIRK channel for efficient channel activation

**DOI:** 10.1371/journal.pone.0204447

**Published:** 2018-09-21

**Authors:** Michihiro Tateyama, Yoshihiro Kubo

**Affiliations:** 1 Division of Biophysics and Neurobiology, Department of Molecular and Cellular Physiology, National Institute for Physiological Sciences, Okazaki, Japan; 2 Department of Physiological Sciences, School of Life Science, SOKENDAI (The Graduate University for Advanced Studies), Hayama, Japan; University of Texas Health Science Center, UNITED STATES

## Abstract

G protein-gated inwardly rectifying K^+^ (GIRK) channel regulates cellular excitability upon activation of Gi/o-coupled receptors. In Gi/o-coupled muscarinic M_2_R, the intracellular third loop (i3) is known as a key domain for Gi/o coupling, because replacement of i3 of Gq-coupled muscarinic M_1_R with that of M_2_R enables the chimeric receptor (MC9) to activate the GIRK channel. In the present study, we showed that MC9, but not M_1_R, co-localizes with the GIRK channel and Gα_i1_ by Förster resonance energy transfer (FRET) analysis. When M_1_R was forced to stay adjacent to the channel through ligation with short linkers, M_1_R activated the GIRK channel. FRET analysis further suggested that the efficacy of channel activation is correlated with the linker length between M_1_R and the GIRK channel. The results show that co-localization is an important factor for activating the GIRK channel. In contrast, for MC9 and M_2_R, the GIRK channel was activated even when they were connected by long linkers, suggesting the formation of a molecular complex even in the absence of a linker. We also observed that replacement of 13 amino acid residues at the N-terminal end of i3 of MC9 with those of M_1_R impaired the co-localization with the GIRK channel as well as channel activation. These results show that localization of the receptor near the GIRK channel is a key factor in efficiently activating the channel and that the N-terminal end of i3 of M_2_R plays an important role in co-localization.

## Introduction

G protein-gated inwardly rectifying potassium (GIRK) channel is a key protein that regulates cellular excitability and is activated by interacting with free Gβγ released from the heterotrimeric Gαβγ complex upon the activation of G protein-coupled receptors (GPCRs) [1]. The conformational rearrangement between the GIRK channel and Gβγ has been observed upon the receptor activation on the living cell membrane by Förster or bioluminescence resonance energy transfer analyses (FRET or BRET, respectively) [2, 3] and the GIRK-Gβγ complex formation was demonstrated by structural analyses [4]. The GIRK channel is also known to bind to Gα_i_ and Gα_o_ in their resting and active forms [5–8], suggesting that Gβγ released from Gα_i_ or Gα_o_ immediately interacts with and activates the GIRK channel. In addition, several Gi/o-coupled receptors (Gi/o-Rs) have been reported to localize near Gi/o by FRET and BRET analyses [9–11]. Therefore, Gi/o-Rs, Gi/o and the GIRK channel have been suggested to form a ternary pre-signaling complex to effectively activate the GIRK channel.

Gi/o-Rs activate the GIRK channel through releasing Gβγ from pertussis toxin (PTX)-sensitive Gα_i/o_ [1, 12]. When the receptors are highly expressed, the GIRK channel is activated by receptors that stimulate PTX-resistant G protein, such as the Gq-coupled muscarinic receptors (M_1_R and M_3_R) or Gs-coupled adrenergic receptors (β1-AR and β2-AR) [13, 14]. When surface expression of M_1_R is very high, the chance of M_1_R-Gi/o coupling may increase [13] and/or the distance between M_1_R and GIRK channel may be shortened, which could allow Gβγ released from Gq to activate the channel. Interestingly, Gs-coupled β2-AR has been reported to functionally and physically interact with GIRK channels [15, 16]. In addition, the rate of GIRK channel activation was thought to correlate with the co-localization of Gi/o-Rs with the channel [17]. We thought that the distance between GPCR and the GIRK channel is a critical factor for effective channel activation.

In this study, we first showed by FRET analyses that a chimeric receptor of M_1_R and M_2_R (MC9) [18], which activates the GIRK channel [19], locates adjacent to the channel and Gα_i1_ in transfected HEK293T cells. Next, we examined whether Gq-coupled M_1_R activates the GIRK channel when these molecules are forced to stay close to each other by ligating them with short linkers. The key amino acid residues in complex formation for activating the GIRK channel were further investigated by analyzing the changes in co-localization of MC9 mutants with the GIRK channel.

## Materials and methods

### Constructs and expression system

The chimeric MC9 receptor was constructed by replacing the last eight residues of the 5th transmembrane (TM) and most of i3 of M_1_R with those of M_2_R (M_2_R-i3) as previously reported [18]. YFP was fused at the C-tail of M_1_R, M_2_R, and MC9 (M_1_R-YFP, M_2_R-YFP, and MC9-YFP, respectively). To control the subunit composition of the GIRK channel, GIRK1 and GIRK2 were ligated (GIRK1/2) [20], by a glycine-rich linker of 34 amino acids (a.a.) (see S1 Method). For FRET analysis, CFP was attached to the C-tail of GIRK1/2 (GIRK1/2-CFP). Fluorescent protein (FP) was inserted into mouse Gα_i1_ between Ala121 and Glu122 (Gα_i1_-FP) with a short linker (A121-SGGGS-V2…K212-SGGGS-E122) [21] or into mouse Gα_q_ between Phe123 and Glu124 with the SGGGS linker. M_1_R-YFP and MC9-YFP were ligated to GIRK1/2 or GIRK1/2-CFP by junctional glycine-rich linkers, with varying lengths of 34, 100, 265, and 535 a.a. residues (S1 Method). The chimeric Gα_qi5_ was constructed by replacing the last five residues of Gα_q_ with those of Gα_i1_ [22]. Each construct was subcloned into the pcDNA3.1(-) expression vector and the DNA sequence was confirmed. The cDNA construct of CFP fused with the PH domain (CFP-PH) was kindly gifted from Dr. Jalink [23]. HEK293T cells were transfected with the plasmid DNAs (μg for 4 x 10^4^ cells) of M_1_R-YFP (0.8), MC9-YFP constructs (0.8), M_2_R-YFP (1.2), GIRK1/2 (0.7), GIRK1/2-CFP (1.2), Gα-FPs (1.1), Gα_qi5_ (1.0), Gβ_1_ (0.8), Gγ_2_ (0.5), M_1_R, MC9 or M_2_R tandem constructs (1.2), CFP-PH (1.1) or prestin-YFP (1.0) [24] using LipofectAMINE 2000 (1 μL, Invitrogen, Carlsbad, CA, USA). The transfected cells were seeded onto cover glasses or glass bottom dishes 8–24 h after the transfection, as previously reported [25]. G protein subunits were not additionally transfected for the electrophysiological experiments and FRET analysis between receptor-YFPs and GIRK1/2-CFP, in order to analyze the interaction between receptor and endogenous Gi/o. Experiments were carried out 24–72 h after transfection. For PTX treatment, the cells were cultured with PTX (300 ng/mL) for more than 16 h and then the experiments were performed.

### Electrophysiology

Macroscopic membrane currents were recorded from cells expressing fluorescent constructs by the whole cell patch clamp technique using Axopatch 200B amplifiers, Digidata1332A, and pClamp 9 software (Molecular Devices, San Jose, CA, USA). After establishing the whole cell configuration, the cell was held at -80 mV and ramp pulses (from -120 to +40 mV for 400 s) were applied every 5 s. The bath solution was composed of the following: 140 mM NaCl, 4 mM KCl, 1 mM CaCl_2_, 0.3 mM MgCl_2_, 10 mM HEPES (pH 7.4 adjusted with NaOH). The internal solution was composed of: 130 mM KCl, 5 mM Na_2_-ATP, 3 mM EGTA, 0.1 mM CaCl_2_, 10 mM HEPES, 4 mM MgCl_2_, 0.3 mM GTP-Li_3_ (pH 7.3 adjusted with KOH). An agonist of muscarinic receptors, oxotremorine M (oxo-M), was applied by using fast perfusion system (VC-77SP, Warner Instruments, Hamden, CT, USA) as previously described [26]. To record the GIRK channel current, Na^+^ ions in the bath solution were substituted with K^+^ to enhance the driving force of K^+^ and oxo-M was applied in 140 mM K^+^ bath solution. The average of the holding current amplitude for 100 ms before applying the ramp pulse was measured and then normalized to the cell capacitance to calculate the current density (I). The basal current density (I_0_) before application of oxo-M (10 μM) was subtracted from the maximum value (I_max_) after agonist application to evaluate the agonist-induced current density (ΔI_max_). To analyze the decay of the GIRK current, the ratio of the agonist-induced current density before washout of the agonist (ΔI_last_) to (ΔI_max_) was calculated. We also analyzed the onset of GIRK channel activation, with the application of oxo-M for 5 s controlled by the combination of Clampex9 and VC-77SP. The time to half-maximum current amplitude (t_1/2_) was then measured for each trace.

### Imaging and FRET analysis

Fluorescent images were obtained from cells expressing FP-fused constructs using a total internal reflection fluorescence (TIRF) microscope (Olympus, Tokyo, Japan) as previously described [25]. MetaFluor imaging software (Molecular Devices, Sunnyvale, CA, USA) was used to control the excitation of CFP and YFP and to acquire images. For FRET analysis, five images were acquired before and after acceptor bleaching, which was performed by excitation of YFP with a 515-nm laser line for 1 min (emission intensity of YFP was decreased by less than 5% in the bleaching procedure). Averaged emission intensities of CFP (I_CFP_) and YFP (I_YFP_) before and after acceptor bleaching were measured in each cell by subtracting their background intensities and were then normalized to the cell size. To calculate FRET efficiency, the increase in I_CFP_ after acceptor bleaching was normalized to the total I_CFP_ after bleaching in each cell. To make the CFP emission images, five CFP images before and after acceptor bleaching were averaged and their subtraction was performed by using MetaMorph software (ver 6, Molecular Devices).

To evaluate the activation of Gqi5 by MC9 constructs, the intensity of CFP-PH (I_CFP-PH_) was measured under TIRF illumination. Cells expressing the fluorescent constructs were continuously perfused with bath solution by gravity at a rate of approximately 3 mL/min, and various concentrations of oxo-M were applied by changing the perfusion solution. The baseline I_CFP-PH_ was the averages of I_CFP-PH_ measured from 13 images before oxo-M application. The maximal decrease in I_CFP-PH_ upon application of oxo-M (ΔI_CFP-PH_) was normalized to the baseline I_CFP-PH_ in each cell.

### Statistical analysis

All data are expressed as the means ± S.E., with n indicating the number of data. Statistical significance between two groups was examined by unpaired Student’s *t*-test and that between more than two groups was tested by one-way analysis of variance (ANOVA) followed by Tukey’s test. Values of p≤0.05 were considered statistically significant (*:0.01<p≤0.05, **:0.001<p≤0.01, ***:p≤0.001, n.s.:p>0.05). Pearson correlation coefficients were calculated using Microsoft Excel2013.

## Results

### i3 of M_2_R is a key structure in activating and staying adjacent to the GIRK channel

**Fig 1:**
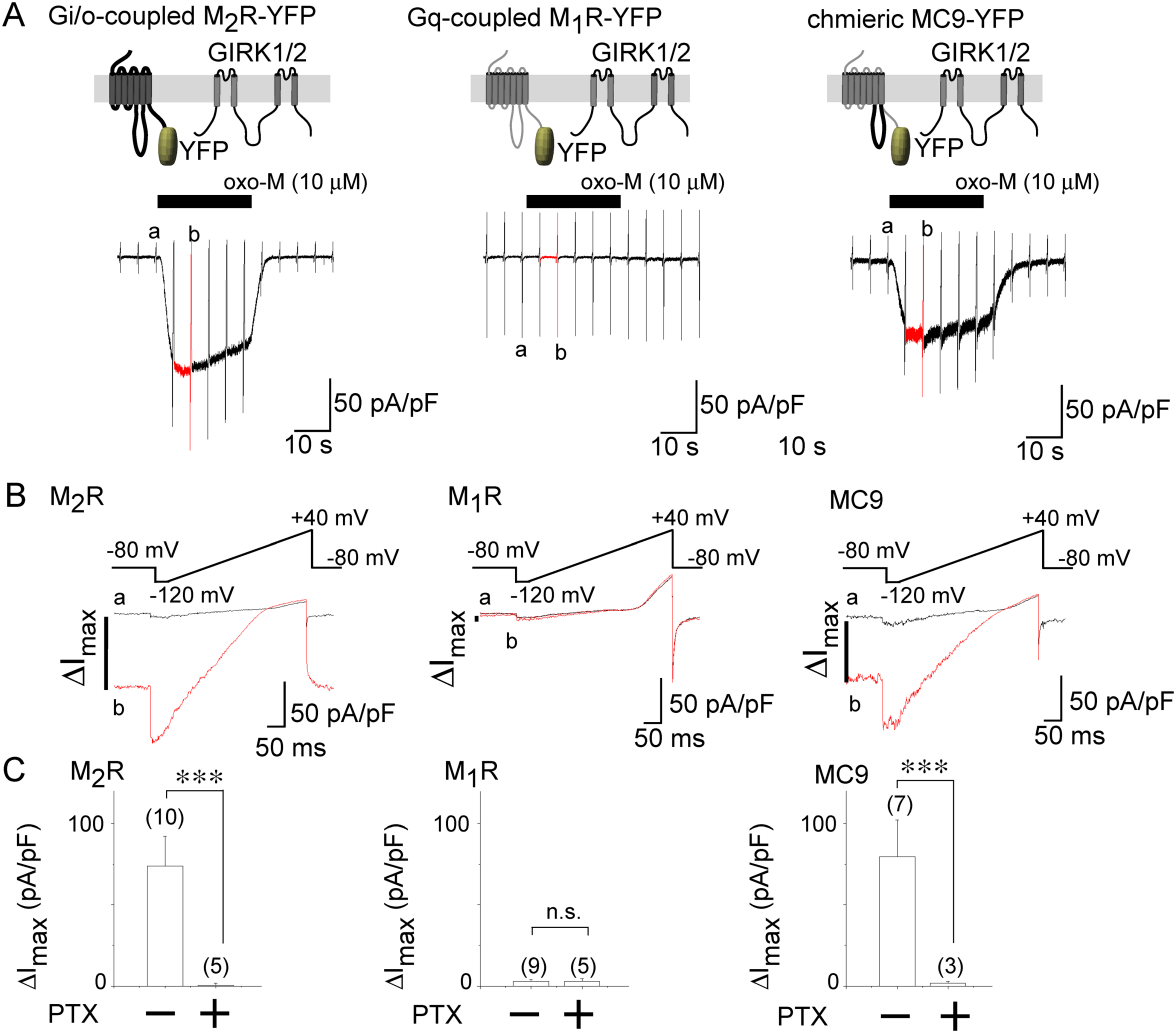
Chimeric MC9 receptor activates GIRK channel. GIRK channel was activated by M_2_R-YFP (left) and MC9-YFP (right) but not by M_1_R-YFP (middle). (A) Schematic diagrams in upper panels depict the tested muscarinic receptors and GIRK1/2 channel. Shown in the middle panels are the current traces recorded in 140 mM KCl bath solution. Cells were held at -80 mV and the ramp pulse (-120 to 40 mV for 400 ms) was applied every 5 s. The black bars on the traces indicate the timing of agonist application. Basal and maximal current amplitude was measured at the holding potential before (a) and during (b, red) oxo-M application. (B) Expanded traces corresponding to “a” (black lines) and “b” (red lines) are shown in middle panels. The ramp pulse protocol is shown above the expanded trace. The agonist-induced current densities at a holding potential of -80 mV (ΔI_max_) was measured. (C) Summary of ΔI_max_ is shown as bars. Numbers of data are shown in parentheses. ***:p≤0.001, n.s.:p>0.05.

**Fig 2:**
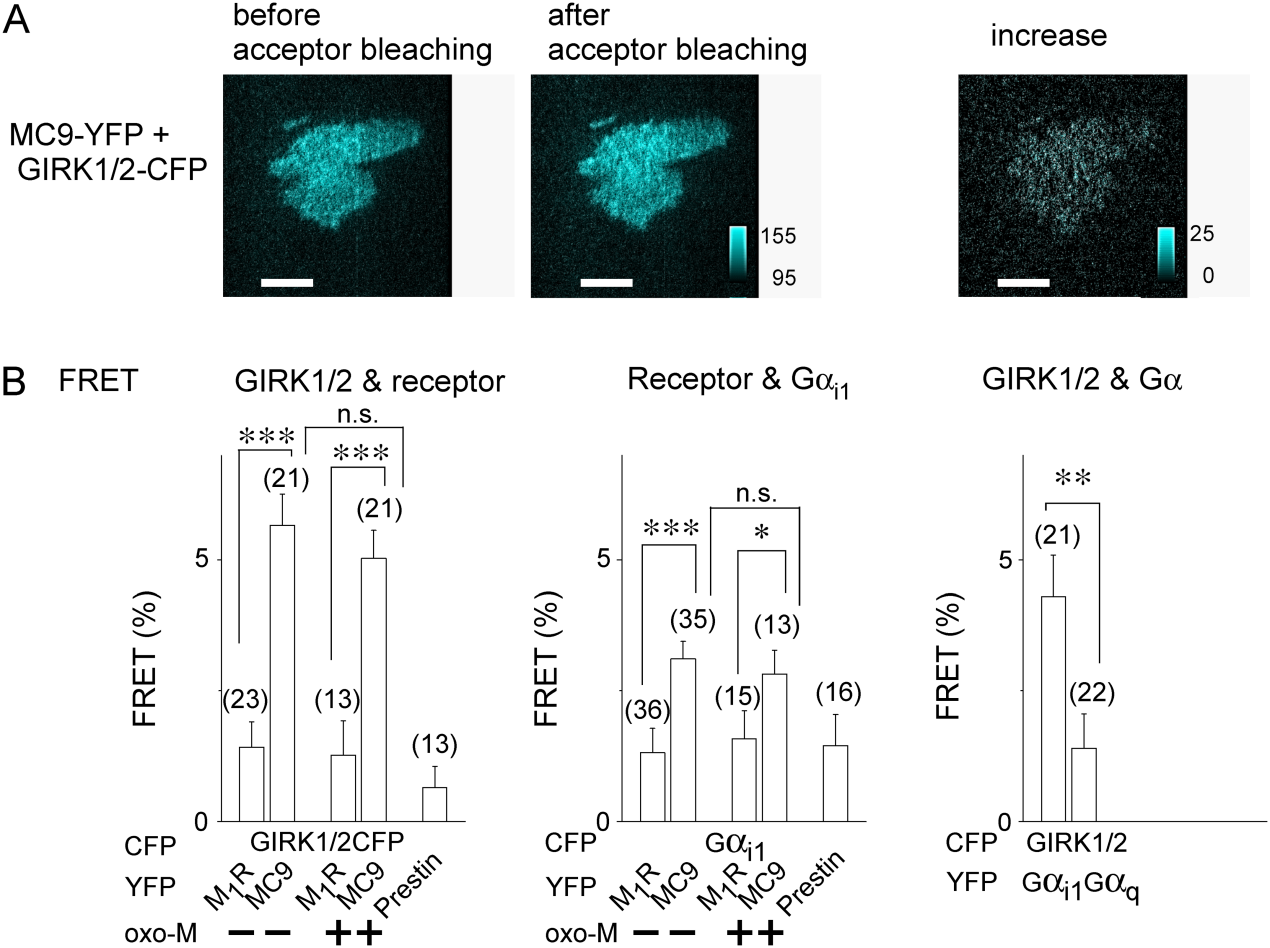
Chimeric MC9 receptor remains in proximity of GIRK channel. (A) Representative CFP emission images before (left panel) and after (middle panel) acceptor bleaching, and their subtraction (right panel). Cells expressing GIRK1/2-CFP and MC9-YFP were excited by 442 nm laser under TIRF illumination. YPF was bleached by exposing strong 515 nm laser line for 1 min. White bars indicate 20 μm and cyan scale bars represent fluorescence intensity. (B) FRET efficiency. FRET efficiency was analyzed in the absence or presence of oxo-M (10 μM) and calculated by the acceptor bleaching method and is shown as bars. Notes below the bar indicate each combination for FRET analysis. Prestin-YFP was thought to reflect non-specific FRET from GIRK-CFP or Gα_i1_-CFP to YFP-tagged membrane protein. Numbers of data are shown in the parentheses. *:0.01<p≤0.05, **:0.001<p≤ 0.01, ***:p≤0.001, n.s.:p>0.05.

Application of an agonist oxo-M increased the amplitude of the inward current density in cells transfected with the GIRK channel and Gi/o-coupled M_2_R, but not with the channel and Gq-coupled M_1_R (Fig 1*A* and 1*B*, left and center panels). The amplitude of the agonist-induced GIRK channel current density (ΔI_max_) was decreased to almost null by treating the cells with PTX (300 ng/mL) (Fig 1*C* left panels), showing that the effect of M_2_R is mediated by the activation of Gi/o. Replacement of the i3 of M_1_R with that of M_2_R (M_2_R-i3) enabled the chimeric receptor (MC9) to activate the GIRK channel, as previously reported [19]. The effect of MC9 was inhibited by PTX treatment (Fig 1*C* right panels) and the speed of current increase induced by the MC9 activation did not differ from that by M_2_R (S1 Fig), indicating that M_2_R-i3 is a critical structure for Gi/o coupling and GIRK channel activation. We then examined whether or not MC9 locates adjacent to the GIRK channel by analyzing FRET from GIRK1/2-CFP to receptor-YFP. In HEK293T cells expressing these constructs, acceptor bleaching resulted in the increases in intensities of CFP (I_CFP_) (Fig 2*A*). Interestingly, the FRET efficiency from GIRK1/2-CFP to MC9-YFP was larger than that to M_1_R-YFP (Fig 2*B* left panels). As the surface expression level of the FP-tagged constructs were not different (S1 Table), MC9 but not M_1_R was suggested to co-localize with the GIRK channel. The FRET efficiency did not change by application of oxo-M (Fig 2*B*), suggesting that activation of receptor does not change the co-localization. We also analyzed the FRET from Gα_i1_-CFP to receptor-YFP in HEK293T cells transfected together with Gβ_1_ and Gγ_2_. The FRET efficiency from Gα_i1_-CFP to MC9-YFP was higher than that to M_1_R-YFP both in the absence and presence of the agonist (Fig 2*B* bottom bars). The results support pre-coupling of MC9 with Gα_i1_ and show that pre-coupling is not changed by MC9 activation. The interaction between GIRK1/2 and Gα_i1_ showed higher FRET efficiency than that between GIRK1/2 and Gα_q_ (Fig 2*B*, right panel), which is consistent with previous reports [5–8]. As a negative control for the FRET analysis, we chose a member of an anion-transporter family, prestin [24], since prestin is not expected to selectively interact with the GIRK channel and G protein. FRET values from CFP tagged constructs to prestin-YFP were similar to those to M_1_R-YFP (Fig 2), suggesting that M_1_R does not specifically interact with GIRK channel and Gα_i1_. Taken together, MC9, GIRK, and Gi/o are adjacent to each other and may form a molecular complex.

### Distance between receptor and GIRK1/2 channel is a key determinant for channel activation

**Fig 3:**
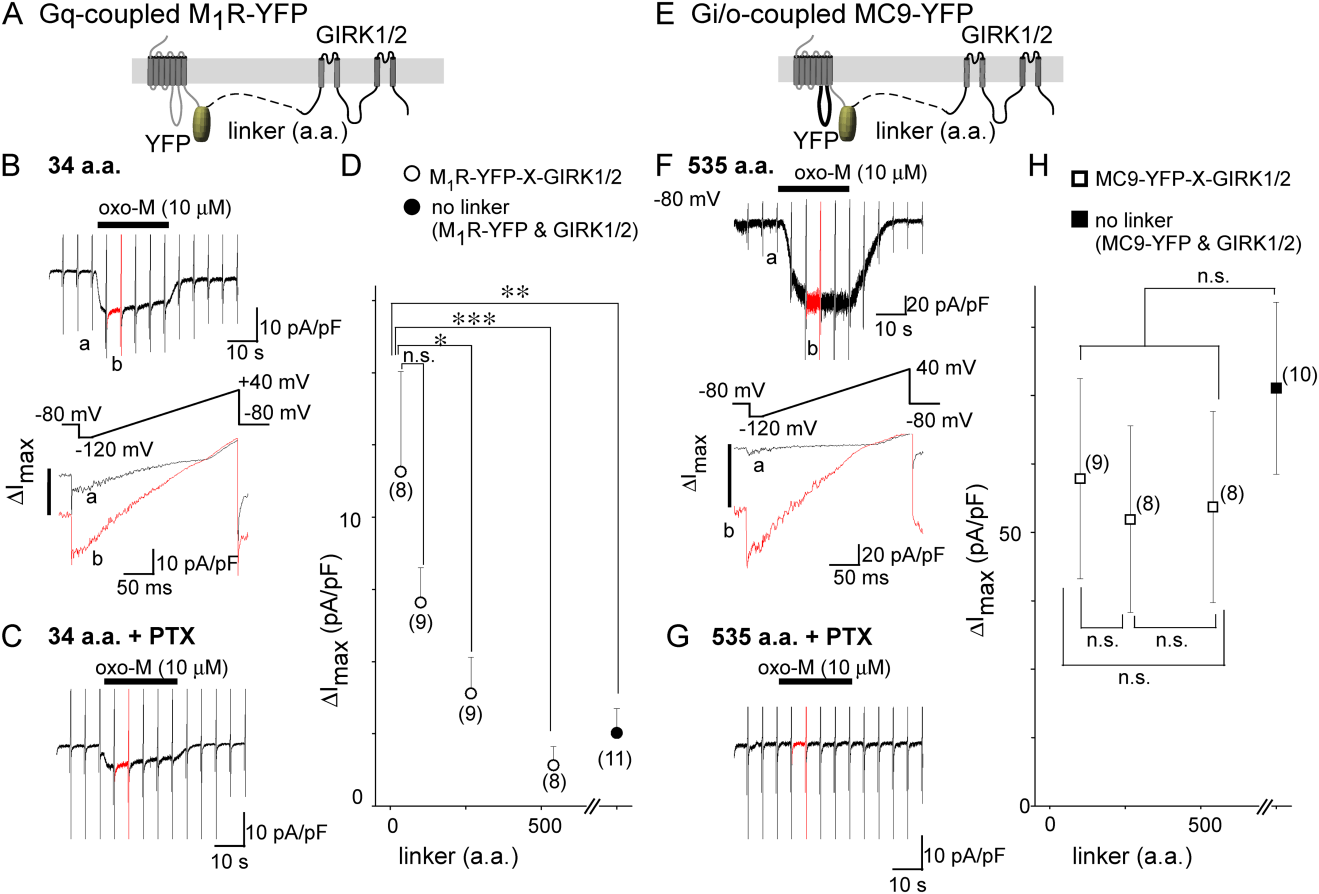
M_1_R activates GIRK channel when they are ligated with short junctional linkers. GIRK channel activation in tandem constructs of M_1_R-YFP (A–D) and of MC9-YFP (E–H). (A) and (E) Schematic diagram of constructs. Shown are the tandem constructs of receptor-YFP and GIRK1/2 channel, which were ligated by junctional linkers. (B) and (F) GIRK channel currents are shown in the upper panel. Whole cell currents were recorded from cells expressing M_1_R-YFP-34-GIRK1/2 (B) or MC9-YFP-535-GIRK1/2 (F) in 140 mM KCl bath solution. Application of oxo-M (10 μM for 25 s) is indicated as black bars on the upper traces. Time scale of current traces corresponding to “a” (before application of oxo-M, black lines) and to “b” (after application of oxo-M, red lines) is expanded and shown in the lower panel. The ramp pulse protocols are shown above the expanded traces in the bottom. (C) and (G) GIRK channel current in the PTX-treated cell. Cells expressing M_1_R-YFP-34-GIRK1/2 (*C*) or MC9-YFP-535-GIRK1/2 (*G*) were treated with PTX (300 ng/mL). (D) and (H) Correlation between ΔI_max_ and the linker length. The amplitudes of agonist-induced GIRK channel current density (ΔI_max_) are plotted as a function of the length of the linker residues. Circles represent ΔI_max_ of the M_1_R constructs (*D*, open circles: tandem constructs, filled circle: no linker). Squares represent ΔI_max_ of the MC9 constructs (*H*, open squares: tandem constructs, filled square: no linker). Numbers of data are shown in the parentheses. *:0.01<p≤0.05, **:0.001<p≤ 0.01, ***:p≤0.001, n.s.:p>0.05.

The FRET analysis raised a question about the functional significance of co-localization. To address this question, Gq-coupled M_1_R was forced to stay adjacent to the GIRK channel by connecting them with a short linker composed of 34 glycine-rich amino acid (a.a.) residues (Fig 3*A*). Application of oxo-M (10 μM) elicited a rapid increase in the amplitude of the inward current in cells transfected with M_1_R-YFP-34-GIRK1/2 (Fig 3*B* red lines). This effect remained after PTX treatment (Fig 3*C*, Table 1), suggesting that Gβγ released from PTX-resistant Gα_q_ activated the channel. The amplitude of the GIRK channel current decreased by approximately 30% at 25 s after application of oxo-M, which may have resulted from negative regulation induced by Gq signaling [27]. When the linker length was elongated, the GIRK channel was not activated; ΔI_max_ was decreased in accordance with the increase in the linker length of the M_1_R tandem constructs (Fig 3*D* open circles). In contrast, the application of oxo-M evoked a rapid increase in the inward current, even when MC9-YFP was connected to GIRK1/2 by a long linker of 535 a.a. residues (Fig 3*E* and 3*F*). The response was totally mediated by PTX-sensitive Gi/o (Fig 3*G*, Table 1). Similar results were observed when the linker length was 100 or 265 a.a. (Fig 3*H*, Table 1), indicating that the effect of MC9 does not change by the linker length. ΔI_max_ in the MC9 tandem constructs were 30% smaller than that in non-linker combination, but the difference was not statistically significant (Fig 3*H*). The ligation of the receptor and the GIRK channel may have a slight inhibitory effect on the expression and/or activation of the GIRK channel. These results suggest that MC9, but not M_1_R, is in proximity of the GIRK channel, even when the linker is long, presumably by forming a molecular complex.

**Table 1:** Effects of PTX treatment on the GIRK channel activation of M_1_R-YFP and MC9-YFP tandem constructs.

Constructs	ΔI_max_ (pA/pF) PTX (-)	ΔI_max_ (pA/pF) PTX (+)
M_1_R-YFP-34-GIRK1/2	11.3 ± 2.5 (11)	8.5 ± 2.2 (12) ^n.s.^
M_1_R-YFP-100-GIRK1/2	9.9 ± 1.5 (16)	8.0 ± 1.5 (15) ^n.s.^
M_1_R-YFP-265-GIRK1/2	6.5 ± 1.2 (12)	4.4 ± 1.7 (7) ^n.s.^
MC9-YFP-100-GIRK1/2	43.7 ± 12.3 (12)	0.4 ± 0.2 (3) **
MC9-YFP-265-GIRK1/2	34.6 ± 10.7 (11)	0.4 ± 0.3 (3) **
MC9-YFP-535-GIRK1/2	36.0 ± 10.4 (9)	0.1 ± 0.1 (3) **

Activation of GIRK channel was evaluated as the amplitude of the agonist-induced increase in the inward current density (ΔI_max_) at the holding potential of -80 mV. Numbers of cells are indicated in parentheses.

**:0.001<p≤ 0.01,

n.s.: p>0.05

**Fig 4:**
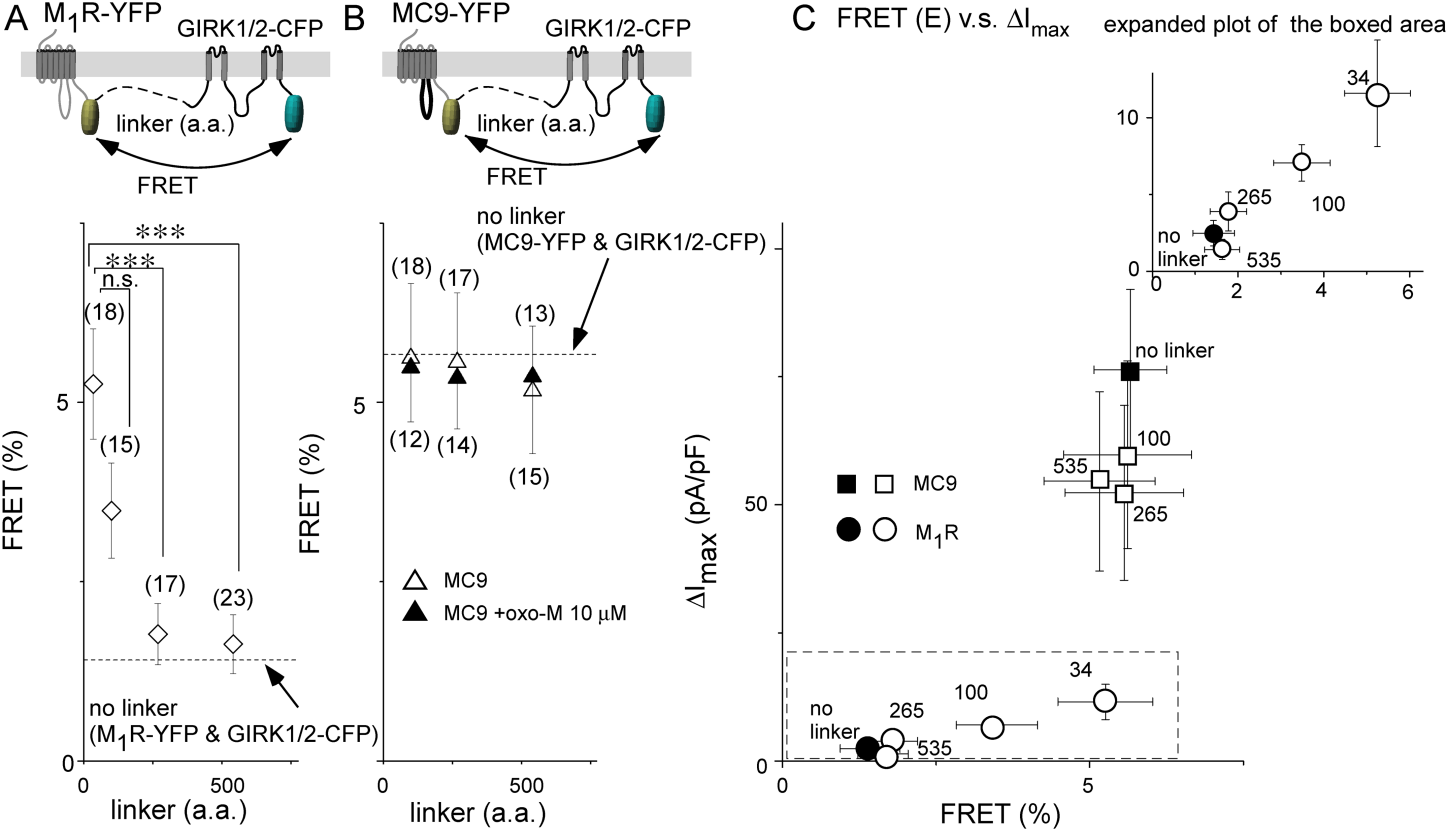
Distance from M_1_R to GIRK channel correlates with efficacy of channel activation. (A) and (B) FRET analyses. Schematic diagrams in the top panel illustrate the tandem construct for the FRET analysis. Symbols represent FRET efficiency in each tandem construct in the absence of oxo-M (A, open diamonds for M_1_R tandem constructs, B, open triangles for MC9 constructs) or presence of oxo-M (B, filled triangles for MC9 constructs). Dashed lines indicate FRET efficiency when the receptor-YFP and GIRK1/2-CFP were co-expressed (no linker, data form Fig 2*B*). Numbers of data are shown in the parentheses. ***:p≤0.001, n.s.:p>0.05 (C) Correlation between ΔI_max_ and FRET efficiency. ΔI_max_ is plotted as a function of the FRET efficiency for each tandem construct (open symbols) or each combination (no linker, filled symbols). The number of junctional linker residues is indicated on each symbol. The results of M_1_R tandem constructs are expanded in the inset to highlight the correlation between FRET efficiency and the GIRK channel activation.

Next, the FRET efficiency between GIRK1/2-CFP and M_1_R- or MC9-YFP was measured for each tandem construct. FRET efficiency in the M_1_R tandem construct was decreased in accordance with the increase in the linker length (Fig 4*A* open diamonds), whereas that in the MC9 construct was not changed by the change of the linker length in the presence or absence of the agonist (Fig 4*B* triangles). For M_1_R, FRET efficiency was well-correlated with ΔI_max_ (Fig 4*C*, Pearson correlation coefficient is 0.98). The results suggest that receptor-GIRK signaling tightly depends on their distance. For MC9, the FRET efficiency and ΔI_max_ did not change when the linker length was changed (Fig 4*C*, Pearson correlation coefficient is 0.53). These results support that MC9, but not M_1_R, forms a complex with the GIRK channel for efficient channel activation.

### M_2_R forms a complex with GIRK channel

**Fig 5:**
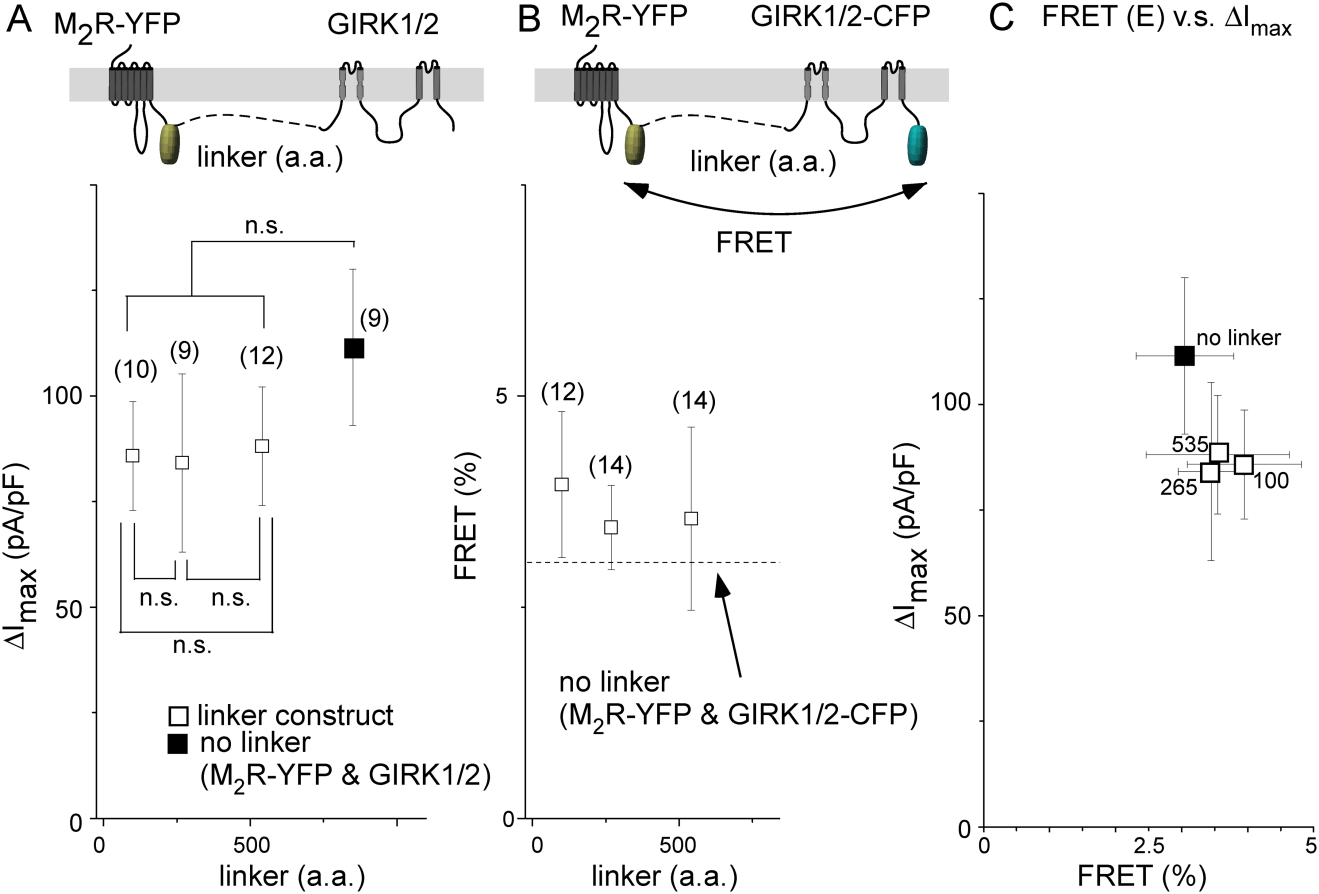
M_2_R forms a molecular complex with GIRK channel. (A) GIRK current density. Schematic diagram in the top panel illustrates the tandem construct. Symbols represent the amplitude of the agonist-induced GIRK current density (ΔI_max_) recorded from cells transfected with M_2_R-YFP tandem constructs (open squares) or co-transfected with M_2_R-YFP and GIRK1/2 (no linker, filled square). Numbers of data are shown in the parentheses. n.s.:p>0.05 (B) FRET analysis. Schematic diagram in the top panel illustrates the construct for the FRET analysis. Symbols represent FRET efficiency in each tandem construct (open squares). The dashed line indicates FRET efficiency when the M_2_R-YFP and GIRK1/2-CFP were co-expressed (no linker). Numbers of data are shown in the parentheses. (C) Correlation between ΔI_max_ and FRET efficiency. ΔI_max_ is plotted as a function of FRET efficiency for each tandem construct (open squares) or a combination with no linker (filled square). The number of junctional linker residues is indicated on each symbol.

M_2_R-i3 is the key structure in complex formation with the GIRK channel and Gi/o (Figs 1 and 2). Thus, the results of the M_2_R tandem constructs connected to the GIRK channel by various linker residues were expected to be similar to those observed in MC9 constructs (Figs 3*H* and 4*B*). As expected, ΔI_max_ as well as FRET efficiency were not changed with the increases in the linker length (Fig 5*A* and 5*B*) and ΔI_max_ was not positively correlated with FRET efficiency (Fig 5*C*, Pearson correlation coefficient is -0.79). GIRK current increase induced by M_2_R was completely inhibited by the PTX treatment (Table 2), indicating that the effects were mediated by the activation of Gi/o in the M_2_R tandem constructs. The FRET efficiency from GIRK to M_2_R was smaller than that to MC9, which may be due to the difference in the C-terminal tail of the receptor at which YFP is tagged. Gi/o was suggested to be located in proximity to M_2_R as well as the GIRK channel; the FRET efficiency from Gα_i1_-CFP to M_2_R-YFP was 2.8 ± 0.5 (n = 12, p = 0.001 compared to FRET from Gα_i1_-CFP to prestin-YFP in Fig 2*B*). Taken together, these results indicate that M_2_R, Gi/o, and the GIRK channel form a molecular complex.

**Table 2:** Inhibitory effects of PTX treatment on the GIRK channel activation by M_2_R-YFP tandem constructs.

construct	ΔI_max_ (pA/pF) PTX (-)	ΔI_max_ (pA/pF) PTX (+)
M_2_R-YFP-100-GIRK1/2	62.0 ± 7.7 (12)	0.9 ± 0.4 (3) ***
M_2_R -YFP-265-GIRK1/2	49.0 ± 11.3 (7)	1.2 ± 0.5 (3) **
M_2_R -YFP-535-GIRK1/2	49.3 ± 10.0 (12)	0.4 ± 0.3 (3) ***

Activation of GIRK channel was evaluated as the amplitude of the agonist-induced increase in the inward current density (ΔI_max_) at the holding potential of -80 mV. Numbers of cells are indicated in parentheses.

***:p≤0.001,

**:0.001<p≤ 0.01

### Dual roles of M_2_R-i3 in the activation of and co-localization with GIRK

**Fig 6:**
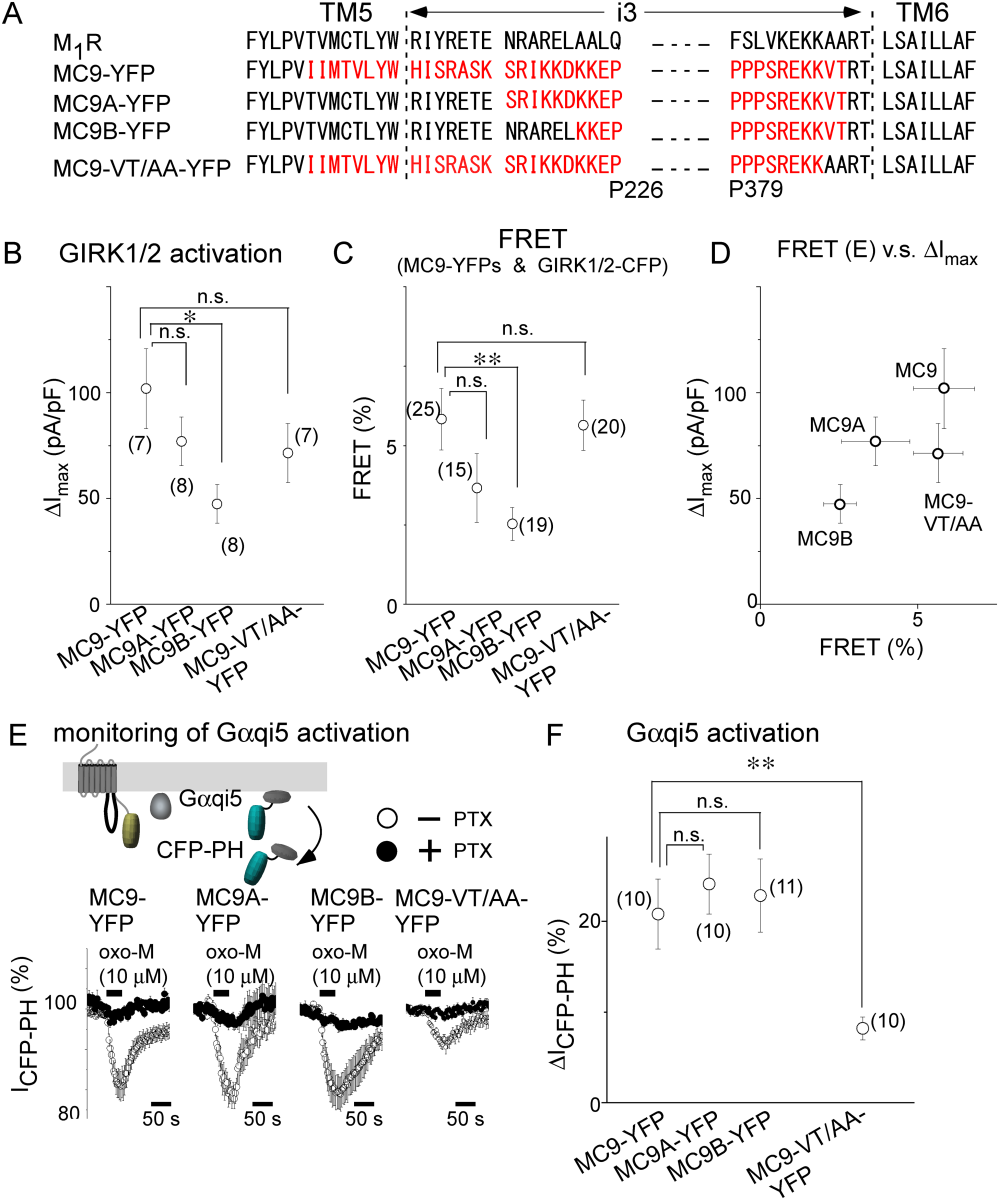
N-Terminal end of M_2_R-i3 plays important roles in the activation of and co-localization with GIRK channel. (A) Amino acid sequences of i3 of mutant and chimeric constructs of MC9-YFP (black: M_1_R sequences, red: M_2_R sequences). (B) Summary of ΔI_max_. Circles represent agonist-induced GIRK channel current density (ΔI_max_) upon application of oxo-M (10 μM). (C) Summary of FRET efficiency between MC9-YFP constructs and GIRK1/2-CFP in the absence of the agonist. FRET efficiency was calculated by acceptor bleaching (see experimental procedures). Fluorescent intensities of YFP and CFP in each combination under TIRF illumination were measured and are summarized in S2 Table. (D) Correlation between ΔI_max_ and FRET efficiency. ΔI_max_ is plotted as a function of FRET efficiency for each MC9-YFP construct (open circles). (E) Activation of Gqi5 by MC9-YFP constructs. Schematic diagram of monitoring of the activation of Gqi5 is shown in the upper panel. Under TIRF illumination, application of oxo-M decreased the intensity of CFP tethered at the PH domain (I_CFP-PH_), as shown in lower traces in the absence of PTX treatment (open circles). The inhibitory effects of PTX (filled circles) are summarized in Table 3. (F) Summary of agonist-induced decrease in I_CFP-PH_ (ΔI_CFP-PH_). Circles represent ΔI_CFP-PH_ upon application of oxo-M (10 μM). Numbers of experiments are indicated in parentheses. *:0.01<p≤0.05, **:0.001<p≤ 0.01, n.s.:p>0.05.

We then evaluated the residues in M_2_R-i3 responsible for activation of and co-localization with the GIRK channel and activation of Gi/o. It was previously reported that most of the long M_2_R-i3 is not necessary for Gi/o coupling [28]. Indeed, the replacement of the long chain in the middle of M_2_R-i3 (K221-P379) with SGGGS did not decrease ΔI_max_ and the FRET efficiency (ΔI_max_ = 75.1 ± 12.5 pA/pF, n = 10; FRET = 5.5 ± 0.9%, n = 15, cf Figs 1 and 2). Thus, the proximal N- and distal C-terminal residues of M_2_R-i3 may be critical regions; the charged residues at the proximal N-terminal end of M_2_R-i3 as well as Val and Thr residues at the distal C-terminal end have been suggested to play important roles in Gi/o coupling [19, 28, 29]. Residues at the N-terminal end were replaced with those of M_1_R (MC9A-YFP and MC9B-YFP, Fig 6*A*) and also double mutations (V387A/T388A) were introduced at the C-terminal end. The emission intensities of YFP (I_YFP_) fused to these constructs under TIRF illumination did not differ (I_YFP_ in S2 and S3 Tables), indicating that their surface expression levels were similar.

We first measured ΔI_max_ and FRET from GIRK1/2-CFP to chimeric and mutant receptor-YFP. Although it was not statistically significant, ΔI_max_ in VT/AA mutants was 30% smaller than that in MC9-YFP (Fig 6*B*), possibly due to the impairment of the activation of Gi/o. Interestingly, ΔI_max_ in MC9B-YFP was significantly smaller than that in MC9-YFP (Fig 6*B*). Similarly, the FRET efficiency from GIRK1/2-CFP to MC9B-YFP, but not to MC9-VT/AA-YFP, was smaller than that to MC9-YFP (Fig 6*C*). ΔI_max_ and FRET values appeared to be correlated in MC9-YFP chimeric constructs (Fig 6*D*): Pearson correlation coefficient is 0.79 (+VT/AA) and 0.97 (-VT/AA). Next, we examined the effect of the chimeric and double mutation on the Gi/o activation by using Gα_qi5_, which is activated by Gi/o-Rs and stimulates Gq-phospholipase C (PLC) signaling [22]. As the last five a.a. residues of Gα_qi5_ are derived from Gα_i1_ and include the ADP-ribosylation site, Gα_qi5_ is inhibited by PTX. The activity of Gα_qi5_ signaling was monitored as the decrease in fluorescent intensity of the CFP-tagged PH domain (CFP-PH) under TIRF illumination (Fig 6*E* upper panel). Upon PIP_2_ hydrolysis, CFP-PH translocates from the membrane into the cytosol, where TIRF illumination cannot reach [23, 26], resulting in a decrease in the fluorescent intensity of CFP-PH (I_CFP-PH_). Indeed, activation of MC9-YFP constructs decreased I_CFP-PH_ in the presence of Gα_qi5_ (Fig 6*E*, lower left open circles). The amplitude of the decrease in I_CFP-PH_ (ΔI_CFP-PH_) was almost null when cells were treated with PTX (Fig 6*E* filled circles and Table 3), suggesting that ΔI_CFP-PH_ may reflect the efficacy of the receptor to activate Gi/o. As previously reported in studies of M_2_R [28, 29], VT/AA attenuated Gα_qi5_ activation: ΔI_CFP-PH_ in the VT/AA mutant was smaller than that in MC9-YFP (Fig 6*F*). In contrast, ΔI_CFP-PH_ was not changed by chimeric mutations of MC9A and MC9B (Fig 6*F*). These results suggest a possibility that all MC9 constructs are equally not co-localized with Gα_qi5_ and/or PLC. Another possibility is that there is a difference in the extent of co-localization with Gα_qi5_ between the MC9, MC9A and MC9B, and that the difference of the co-localization does not change the extent of PLC activation. If this is the case, co-localization might not be critical for the PLC activation, highlighting the significance of co-localization for the GIRK channel activation. We also analyzed FRET from Gα_i1_-CFP to MC9B-YFP and found that co-localization with Gα_i1_ appeared to be attenuated, but the difference was not significant (S3 Table). Taken together, at least the 13 a.a. residues at the N-terminal end of M_2_R-i3 play important roles in co-localization with the GIRK channel as well as channel activation, while Val and Thr residues at the C-end are required for Gq signaling by Gα_qi5_ activation.

**Table 3:** Inhibitory effects of PTX treatment on the activation of Gqi5 or GIRK channel currents by MC9-YFP constructs.

GIRK channel activation	ΔI_max_ (pA/pF) PTX (-)	ΔI_max_ (pA/pF) PTX (+)
MC9-YFP	101.9 ± 18.9 (7)	1.3 ± 0.9 (3) ***
MC9A-YFP	77.0 ± 11.5 (8)	0.1 ± 0.6 (3) ***
MC9B-YFP	47.4 ± 9.1 (8)	2.5 ± 1.7 (3) ***
MC9-YFP-VT/AA	71.5 ± 14.0 (7)	1.7 ± 0.6 (3) ***
Gqi5 activation	ΔI_CFP-PH_ (%) PTX (-)	ΔI_CFP-PH_ (%) PTX (+)
MC9-YFP	20.8 ± 3.8 (10)	3.6 ± 1.0 (6) ***
MC9A-YFP	24.1 ± 3.3 (10)	5.9 ± 1.0 (4) ***
MC9B-YFP	22.9 ± 4.0 (11)	5.5 ± 1.2 (5) **
MC9-YFP-VT/AA	8.2 ± 1.2 (10)	3.7 ± 0.3 (7) **

Activation of GIRK channel was evaluated as the amplitude of the agonist-induced increase in the inward current density (ΔI_max_) at the holding potential of -80 mV. Activation of Gqi5 was evaluated as the decrease in the intensity of CFP-PH (ΔI_CFP-PH_) under TIRF illumination. Numbers of cells are indicated in parentheses.

***:p≤0.001,

**:0.001<p≤0.01

### Coupling properties of MC9B and MC9-VT/AA

**Fig 7:**
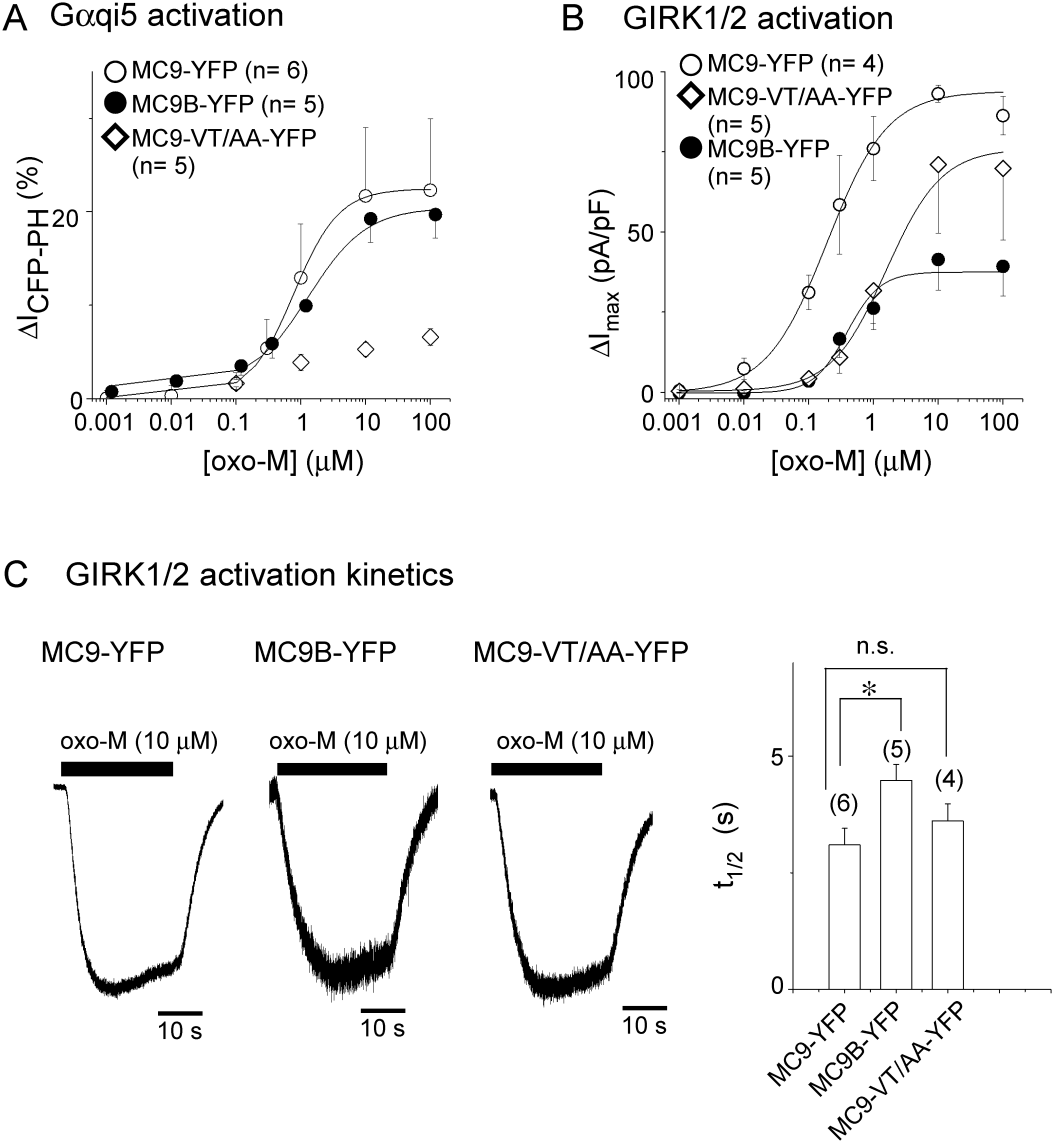
Properties of MC9B and MC9-VT/AA receptors. (A) Oxo-M concentration-ΔI_CFP-PH_ relationships. I_CFP-PH_ was decreased upon application of various concentrations of oxo-M in cells transfected with MC9-YFP constructs. ΔI_CFP-PH_ is plotted as a function of oxo-M concentration (MC9-YFP, open circles; MC9B-YFP, filled circles; MC9-VT/AA-YFP, diamonds). EC_50_ value is 0.82 ± 0.20 μM (n = 6) for MC9-YFP and 0.99 ± 0.32 μM (n = 5) for MC9B-YFP (p>0.05). EC_50_ was not calculated for MC9-VT/AA-YFP because of the small changes of I_CFP-PH_. (B) Oxo-M concentration-ΔI_max_ relationships. The amplitude of the GIRK channel current density (ΔI_max_) induced by various concentrations of oxo-M is plotted as a function of oxo-M concentration (symbols are indicated in A). EC_50_ value is 0.22 ± 0.06 μM (n = 4) for MC9-YFP, 0.59 ± 0.10 μM (n = 5) for MC9B-YFP (p>0.05, vs MC9-YFP) and 1.16 ± 0.13 μM (n = 5) for MC9-VT/AA-YFP (p≤0.001 vs MC9-YFP). (C) Activation kinetics of GIRK channel current upon oxo-M application. Traces represent the GIRK channel current recorded from cells expressing MC9-YFP constructs and GIRK1/2. The application of oxo-M (10 μM) was controlled by a rapid perfusion system. Bars in right represent the half time to maximum (t_1/2_). Numbers of experiments are indicated in parentheses. *:0.01<p≤0.05, n.s.:p > 0.05.

The oxo-M concentration dependence of MC9B-YFP and MC9-VT/AA-YFP was then investigated. Examination of the concentration-ΔI_CFP-PH_ relationship (Fig 7*A*) revealed that EC_50_ did not differ between MC9 and MC9B. In the case of VT/AA mutant, 100 μM oxo-M was not the saturating concentration which made the estimation of EC_50_ impossible and support that VT/AA mutations impaired the Gα_qi5_ activation. The concentration-ΔI_max_ of MC9-VT/AA-YFP showed that EC_50_ of the mutant was 5-fold larger than that of MC9-YFP (Fig 7*B*). The lower affinity may represent impaired Gi/o activation. In the case of MC9B, the EC_50_ was 2-fold larger than that of MC9 (Fig 7*B* filled circles). These results indicate that the coupling efficacy between MC9B and the GIRK channel was reduced. As the speed of the GIRK channel activation is another index of signaling efficacy, we analyzed the activation speed by using a motor-driven fast-perfusion system to control agonist application. The time to reach the half-maximal current (t_1/2_) upon activation of MC9B was longer than that of MC9 (Fig 7*C*), consistent with the results showing that GIRK and MC9B are distant from each other [17]. Taken together, the N- and C-terminal ends of M_2_R-i3 were shown to have different roles in receptor-GIRK signaling.

## Discussion

We showed that M_2_R-i3 enables the chimeric MC9 to locate adjacent to Gα_i1_ and the GIRK channel, that Gq-coupled M_1_R activates the GIRK channel when it is in proximity to the channel, and that the distance between the receptor and GIRK channel is a key determinant in effective channel activation.

### Interaction between Gi/o-coupled muscarinic receptors, GIRK and Gi/o

Localization of MC9 or M_2_R in proximity to the GIRK channel was shown by FRET analysis and experiments using tandem constructs (Figs 2–5). These results were consistent with those of the previous FRET study of metabotropic receptor GABA_B_R, Gi/o, and GIRK [10]. As suggested for other Gi/o-Rs [9–11], MC9 and M_2_R are likely to be pre-coupled with Gi/o (Fig 2*B*). Because Gα_i/o_ binds to the GIRK channel either in GTP-bound form or GDP-bound form [5–8, 30], the Gi/o-GIRK coupling is thought to play important roles in the ternary complex formation. In fact, replacement of the helical domain of Gα_q_ with that of Gα_i_ was reported to enable the chimeric G protein to interact with the GIRK channel and the Gq-coupled M_1_R to activate the channel in cells treated with PTX [30]. Therefore, M_2_R and MC9 were suggested to localize near the GIRK channel by pre-coupling with Gi/o. In the case of MC9 chimeric mutants, MC9B showed significant impairment of the activation of and co-localization with the GIRK channel (Fig 6). Although it was not statistically significant in MC9B, the co-localization with Gα_i1_ was attenuated (S3 Table). The lack of statistical significance (MC9B-YFP vs MC9-YFP, S3 Table) was possibly due to the small FRET efficiency between Gα_i1_-CFP and YFP tagged constructs relative to the background level. We think that the proximal N-terminal residues of M_2_R-i3 is a key region for the pre-coupling of M_2_R and MC9 with Gi/o. However, another possibility that the proximal N-terminal residues may play some roles in direct interaction with the GIRK channel cannot be excluded.

FRET efficiency between MC9, Gi/o, and the GIRK channel was not changed by receptor activation (Figs 2*B* and 4*B*). These results were different from those of previous FRET studies, in which agonist-induced increases in the FRET efficiency was shown between Gq-Rs and Gα_q_ [25, 26] or between adenosine receptor A1R and Gi1 [31]. Similar FRET increases have been reported between adrenergic receptors (α2A-AR and β1-AR) and G protein [32, 33]. As revealed in a recent single-molecule imaging study of G proteins and α2A-AR or β1-AR, receptors and G proteins dynamically associate with each other [34]. These reports supported the notion that the activated receptors associate with and activate G protein, which is detected as agonist-induced increases in FRET between receptor and G protein. In contrast, agonist-induced FRET increases were not detected in several Gi/o-Rs, which were suggested to pre-couple with Gi/o [9–11]. These reports are consistent with the results showing that pre-coupling is not changed by receptor activation. Interestingly, an agonist-induced decrease in FRET was observed between the opioid receptor and Gα_i1_ [9]. Therefore, the receptor-G protein interaction may differ depending on the receptor type.

### Difference in efficacy of GIRK channel activation between M_1_R and MC9

M_1_R activates the GIRK channel only when it is in proximity of the channel by linking them with a short linker (Fig 3), but ΔI_max_ upon activation of M_1_R was lower than that of MC9 (Fig 3*D* and 3*H*). As FRET efficiency did not differ between the M_1_R and MC9 constructs ligated with the GIRK channel by a short linker (Fig 4*A* and 4*B*), the relative distance from M_1_R to the channel is likely to be similar to that from MC9. The weak effect of M_1_R on the GIRK channel activation may be because of the low abundance of the endogenous Gα_q_ subunit, as co-expression of Gα_s_ markedly increased the GIRK channel current amplitude for β2-AR [16]. We thus co-expressed Gα_q_ with M_1_R-YFP-100-GIRK1/2, but co-expression failed to increase the amplitude; ΔI_max_ was 6.4 ± 4.5 pA/pF (n = 3). This may be because of the inhibitory effects of Gα_q_ on the GIRK channel [27, 35–37]. In fact, co-expression of Gα_q_ with M_2_R-YFP-100-GIRK1/2 inhibited the effect of M_2_R on the GIRK channel; ΔI_max_ was 3.6 ± 2.5 pA/pF (+Gα_q_, n = 4) and 78.2 ± 15.9 pA/pF (-Gα_q_, n = 5, P = 0.002). Therefore, the weak effect of M_1_R on the GIRK channel activation can be explained by the inhibitory effects of Gα_q_ on the GIRK channel.

### Localization in proximity is a key factor for activating GIRK channel

In MC9-YFP, the EC_50_ of GIRK channel activation was 4-fold lower than that of Gα_qi5_ activation (Fig 7). This can be interpreted as the functional role of receptor-GIRK channel co-localization; localization of MC9 in the proximity to the GIRK channel enhances the efficacy of receptor-GIRK channel signaling through increasing a probability for MC9 to associate with complex of Gi/o and GIRK channel (Fig 2C Gα_i1_ vs. Gα_q_). Interestingly, in the case of MC9B which impaired co-localization with the GIRK channel (Fig 6*C*), the difference of EC_50_ between ΔI_CFP-PH_ and ΔI_max_ was attenuated. The EC_50_ of GIRK channel activation was 2-fold lower than that of Gα_qi5_ activation (Fig 7*A* and 7*B*), which may be results of the decreases in a probability for MC9B to associate with Gi/o-GIRK channel complex. In contrast, mutations at the C-terminal end of M_2_R-i3 (VT/AA mutation) impaired the activation of Gα_qi5_ (Figs 6*F* and 7*A*), consistently with previous reports in which VT/AA mutation of M_2_R disrupted the Gi/o activation [27, 28]. As for the GIRK channel activation, the double mutation did not abolish ΔI_max_ but shifted the concentration-ΔI_max_ relationship rightwards (Fig 7*B*). As co-localization with the GIRK channel was not affected (Fig 6*C*), impairment of Gi/o activation in the double mutant may be compensated by co-localization, which enhances receptor-GIRK signaling.

In conclusion, we showed that the Gi/o-coupled muscarinic receptors co-localize with the GIRK channel and Gα_i1_, which is mediated by at least 13 a.a. residues at the N-terminal end of M_2_R-i3, and that co-localization is another determinant of the efficacy of channel activation.

## Supporting information

S1 Methods Sequences of the glycine rich junctional amino acid residues. (DOCX)

S1 Fig Activation speed of GIRK channel induced by M_2_R and MC9. (DOCX)

S1 Table Fluorescent intensities of FPs fused at receptors, Gα_i1_ or GIRK1/2 and the FRET efficiency under TIRF illumination. (DOCX)

S2 Table FRET efficiency between GIRK1/2 -CFP and MC9-YFP constructs under the TIRF illumination. (DOCX)

S3 Table Fluorescent intensities of Gα_i1_-CFP and MC9-YFP constructs under the TIRF illumination. (DOCX)
