# Supplementary material for: Gi/o-coupled muscarinic receptors co-localize with GIRK channel for efficient channel activation
Source: PLoS One. 2018 Sep 21;13(9):e0204447. doi: 10.1371/journal.pone.0204447 (PMC6150519; doi:10.1371/journal.pone.0204447)
Supplement: S1 Methods — (DOCX) [file pone.0204447.s001.docx]

**S1 Method**

Sequences of the glycine rich junctional amino acid residues are shown below.

34 glycine amino acid residues

TR-(Repeat A)-(GGS)x4-GGGS Repeat A: (GGS)x4-GGTR

100 glycine amino acid residues

SR-(Repeat B)-GGRSTR-(Repeat B)-GGGS Repeat B: (Repeat A)x2-(GGS)x4

265 glycine amino acid residues

SR-(Repeat A)x2-(Repeat C)x2-(Repeat D)x3-(Repeat A)x2-(GGS)x3-GGGS

Repeat C: (GGS)x4-GGRSTR

Repeat D: (Repeat A)x2-(Repeat C)

535 glycine amino acid residues

256 liner-RLNGP-256 linker

GIRK1/2

The Spe1 and BglII restriction sites were introduced before the stop codon of GIRK2 cDNA and before the starting codon of GIRK1 cDNA, respectively, by using PCR reaction with designed primer. The XbaI and BamH1 restriction sites were also introduced at the 5’ and 3’ ends of cDNA for 34 linker residues. After digestion and purification of the PCR fragments, each piece of the fragment was ligated into pcDNA 3.1(-) vector by ligase (Takara, Japan).
