# Supplementary material for: Gi/o-coupled muscarinic receptors co-localize with GIRK channel for efficient channel activation
Source: PLoS One. 2018 Sep 21;13(9):e0204447. doi: 10.1371/journal.pone.0204447 (PMC6150519; doi:10.1371/journal.pone.0204447)
Supplement: S1 Fig — (DOCX) [file pone.0204447.s002.docx]

**S1 Fig 1. Activation speed of GIRK channel induced by M_2_R and MC9**

Shown in the top panel are the schematic diagrams of the tested constructs. Traces represent the GIRK channel currents recorded from cells expressing M_2_R- (left panel) or MC9-YFP and GIRK1/2 (right panel). Application of oxo-M (10 μM) was controlled by a combination of Clampex and perfusion system (black bars on the traces). Bars in right represent the half time to maximum (t_1/2_). There is no statistical significance in the activation speeds.
