# Supplementary material for: Gi/o-coupled muscarinic receptors co-localize with GIRK channel for efficient channel activation
Source: PLoS One. 2018 Sep 21;13(9):e0204447. doi: 10.1371/journal.pone.0204447 (PMC6150519; doi:10.1371/journal.pone.0204447)
Supplement: S1 Table — (DOCX) [file pone.0204447.s003.docx]

**S1 Table. Fluorescent intensities of FPs fused at receptors, Gα_i1_ or GIRK1/2 and the FRET efficiency under TIRF illumination**

|  | I_CFP_ | I_YFP_ | FRET (%) | n |
| --- | --- | --- | --- | --- |
| GIRK1/2-CFP & M_1_R-YFP | 31.3 ± 2.9 | 156.6 ± 22.7 | 1.4 ± 0.5 | 23 |
| GIRK1/2 -CFP & MC9-YFP | 33.5 ± 3.8 | 154.0 ± 20.2 | 5.7 ± 0.6* | 21 |
| Gα_i1_-CFP & M_1_R-YFP | 50.4 ± 3.5 | 140.5 ± 16.9 | 1.5 ± 0.4 | 36 |
| Gα_i1_-CFP & MC9-YFP | 54.3 ± 4.3 | 137.7 ± 12.7 | 3.1 ± 0.3* | 35 |
| GIRK1/2-CFP & Gα_i1_-YFP | 39.5 ± 4.8 | 150.9 ± 11.4 | 4.3 ± 0.8 | 21 |
| GIRK1/2-CFP & Gα_q_-YFP | 38.5 ± 5.4 | 255.5 ± 48.4* | 1.3 ± 0.6* | 22 |

Fluorescent intensity was measured from each cell expressing both fluorescent constructs under the TIRF illumination before and after the photo-bleaching and then normalized by cell size. The normalized intensity of YFP before the photo-bleaching (I_YFP_), that of CFP after the photo-bleaching (I_CFP_) and the FRET efficiency are shown as mean and S.E. Number of cells are indicated as n. These results suggested that the surface expression level of receptor-YFP and GIRK1/2 -CFP were not different among the combinations for the FRET analyses. The membrane localization of Gα_q_-YFP was higher than that of Gα_i1_, which does not contribute to the lower FRET efficiency. *: 0.01<p≤0.05 (Unpaired *t*-test)
