# Supplementary material for: Gi/o-coupled muscarinic receptors co-localize with GIRK channel for efficient channel activation
Source: PLoS One. 2018 Sep 21;13(9):e0204447. doi: 10.1371/journal.pone.0204447 (PMC6150519; doi:10.1371/journal.pone.0204447)
Supplement: S2 Table — (DOCX) [file pone.0204447.s004.docx]

**S2 Table. FRET efficiency between GIRK1/2 -CFP and MC9-YFP constructs under the TIRF illumination**

|  | I_CFP_ | I_YFP_ | FRET (%) | n |
| --- | --- | --- | --- | --- |
| GIRK1/2-CFP & MC9-YFP | 27.0 ± 3.1 | 161.7 ± 20.1 | 5.9 ± 0.6 | 25 |
| GIRK1/2 -CFP & MC9A-YFP | 31.4 ± 3.6 ^n.s.^ | 242.3 ± 33.5 ^n.s.^ | 4.5 ± 1.0 ^n.s.^ | 15 |
| GIRK1/2 -CFP & MC9B-YFP | 36.2 ± 6.0 ^n.s.^ | 212.9 ± 28.0 ^n.s.^ | 2.6 ± 0.6 * | 19 |
| GIRK1/2 -CFP & MC9-YFP-VT/AA | 27.1 ± 2.3 ^n.s.^ | 177.4 ± 22.4 ^n.s.^ | 5.6 ± 0.8 ^n.s.^ | 20 |

Fluorescent intensity was measured from each cell expressing receptor-YFP and GIRK1/2 -CFP under the TIRF illumination before and after the photo-bleaching and then normalized by cell size. The normalized intensity of YFP before the photo-bleaching (I_YFP_), that of CFP after the photo-bleaching (I_CFP_) and the FRET efficiency are shown as mean and S.E. Number of cells are indicated as n. These results suggested that the surface expression level of receptor-YFP and GIRK1/2 -CFP were not different among the tested combinations. *: 0.01<p≤0.05, n.s.: p > 0.05 (v.s. MC9-YFP, Tukey’s test)
