# Supplementary material for: Gi/o-coupled muscarinic receptors co-localize with GIRK channel for efficient channel activation
Source: PLoS One. 2018 Sep 21;13(9):e0204447. doi: 10.1371/journal.pone.0204447 (PMC6150519; doi:10.1371/journal.pone.0204447)
Supplement: S3 Table — (DOCX) [file pone.0204447.s005.docx]

**S3 Table. Fluorescent intensities of Gα_i1_-CFP and MC9-YFP constructs under the TIRF illumination**

|  | I_CFP_ | I_YFP_ | FRET (%) | n |
| --- | --- | --- | --- | --- |
| Gα_i1_-CFP & MC9-YFP | 40.0 ± 4.0 | 136.5 ± 15.4 | 3.4 ± 0.4 | 19 |
| Gα_i1_-CFP & MC9A-YFP | 43.8 ± 2.9 ^n.s.^ | 127.0 ± 13.6 ^n.s.^ | 3.4 ± 0.3 ^n.s.^ | 25 |
| Gα_i1_-CFP & MC9B-YFP | 49.8 ± 3.8 ^n.s.^ | 153.0 ± 16.1 ^n.s.^ | 2.4 ± 0.6 ^n.s.^ | 24 |
| Gα_i1_-CFP & MC9-YFP-VT/AA | 46.3 ± 4.9 ^n.s.^ | 152.6 ± 19.0 ^n.s.^ | 3.8 ± 0.4 ^n.s.^ | 24 |

Fluorescent intensity was measured from each cell expressing receptor-YFP and GIRK1/2 -CFP under the TIRF illumination before and after the photo-bleaching and normalized by cell size. The normalized intensity of YFP before the photo-bleaching (I_YFP_), that of CFP after the photo-bleaching (I_CFP_) and the calculated FRET efficiency are shown as mean and S.E. Number of cells are indicated as n. n.s.: p > 0.05 (v.s. MC9-YFP, Tukey’s test).
